# Peroxisome proliferator-activated receptor gamma as a theragnostic target for mesenchymal-type glioblastoma patients

**DOI:** 10.1038/s12276-020-0413-1

**Published:** 2020-04-13

**Authors:** Tuyen N. M. Hua, Jiwoong Oh, Sohyun Kim, Jayson M. Antonio, Vu T. A. Vo, Jiyeon Om, Jong-Whan Choi, Jeong-Yub Kim, Chan-Woong Jung, Myung-Jin Park, Yangsik Jeong

**Affiliations:** 1 Departments of Biochemistry, Yonsei University, Wonju, Republic of Korea; 2 Departments of Global Medical Science, Yonsei University, Wonju, Republic of Korea; 3 Departments of Mitohormesis Research Center, Yonsei University, Wonju, Republic of Korea; 4 Department of Neurosurgery, Severance Hospital, Yonsei University College of Medicine, Seoul, 03722 Republic of Korea; 5 Department of Physiology, Yonsei University College of Medicine, Seoul, 03722 Republic of Korea; 6 Division of Radiation Biomedical Research, Korea Institute of Radiological and Medical Sciences, Seoul, Republic of Korea; 7 Institutes of Lifestyle Medicine, Yonsei University, Wonju, Republic of Korea; 8 Departments of Mitochondrial Medicine, Yonsei University, Wonju, Republic of Korea; 9 Departments of Nuclear Receptor Research Consortium, Wonju College of Medicine, Yonsei University, Wonju, Gangwon-Do 26426 Republic of Korea

**Keywords:** Cancer stem cells, Cancer

## Abstract

Glioblastomas (GBMs) are characterized by four subtypes, proneural (PN), neural, classical, and mesenchymal (MES) GBMs, and they all have distinct activated signaling pathways. Among the subtypes, PN and MES GBMs show mutually exclusive genetic signatures, and the MES phenotype is, in general, believed to be associated with more aggressive features of GBM: tumor recurrence and drug resistance. Therefore, targeting MES GBMs would improve the overall prognosis of patients with fatal tumors. In this study, we propose peroxisome proliferator-activated receptor gamma (PPARγ) as a potential diagnostic and prognostic biomarker as well as therapeutic target for MES GBM; we used multiple approaches to assess PPARγ, including biostatistics analysis and assessment of preclinical studies. First, we found that PPARγ was exclusively expressed in MES glioblastoma stem cells (GSCs), and ligand activation of endogenous PPARγ suppressed cell growth and stemness in MES GSCs. Further in vivo studies involving orthotopic and heterotopic xenograft mouse models confirmed the therapeutic efficacy of targeting PPARγ; compared to control mice, those that received ligand treatment exhibited longer survival as well as decreased tumor burden. Mechanistically, PPARγ activation suppressed proneural–mesenchymal transition (PMT) by inhibiting the STAT3 signaling pathway. Biostatistical analysis using The Cancer Genomics Atlas (TCGA, *n* = 206) and REMBRANDT (*n* = 329) revealed that PPARγ upregulation is linked to poor overall survival and disease-free survival of GBM patients. Analysis was performed on prospective (*n* = 2) and retrospective (*n* = 6) GBM patient tissues, and we finally confirmed that PPARγ expression was distinctly upregulated in MES GBM. Collectively, this study provides insight into PPARγ as a potential therapeutic target for patients with MES GBM.

## Brain cancer: Nuclear receptor offers diagnostic and therapeutic target

A protein that helps relay signals within tumor cells provides a promising diagnostic biomarker and therapeutic target for the most deadly form of glioblastoma, a brain cancer. A research team in South Korea led by Myung-Jin Park from the Korea Institute of Radiological and Medical Sciences in Seoul and Yangsik Jeong from Yonsei University in Wonju showed that the gene encoding a nuclear receptor protein called PPARγ is exclusively expressed in glioblastoma stem cells taken from patients with the aggressive “mesenchymal” subtype of the disease, but not in stem cells from other glioblastomas with more favorable outcomes. In cell cultures and mouse models, the researchers found that elevated levels of the nuclear receptor promote cancer growth, but activation of PPARγ, either with drugs or by genetic means, has an opposite effect and suppresses tumor proliferation.

## Introduction

Glioblastoma (GBM) is the most malignant and lethal primary brain tumor in humans; GBM yields an extremely poor prognosis and quality of life that is associated with reduced cognitive function^1^. The clinical outcome of this deadly cancer shows a median survival of 15 months and a 2-year postoperative survival rate of 27%. Even GBM patients with well-demarcated tumors who show a favorable initial response to the conventional therapeutic scheme after surgical removal of the tumor lobe eventually relapse with acquired resistance to chemotherapy and/or radiation treatment. Tumor recurrence may be attributed to diverse clinicopathological features involving GBM heterogeneity, which is potentially due to cellular plasticity of stemness^2,3^. Recently, Lee et al.^4^ demonstrated that GBM originates from the subventricular zone of the brain, where normal stem cells acquire driver mutations and become cancer stem cells, which contributes to tumor development as well as therapy resistance^5^. The classification of GBM into four subtypes, proneural (PN), classical, neural, and mesenchymal (MES), is still controversial in the literature, but recent genomics analysis clearly suggests that PN and MES GBMs show mutually exclusive genetic signatures and distinct signaling pathways, in particular metabolic phenotypes, that correspond to the subtypes^6,7^. In addition, PN GBM undergoes a phenotypic shift into the more aggressive MES GBM, the so-called PMT, upon encountering diverse microenvironmental stresses, including inflammation, radiation, or chemotherapy^7,8^. Thus, it is believed that MES GBM may acquire therapeutic resistance to radiation and chemotherapy, which is responsible for tumor recurrence leading to poor prognosis^8,9^. Considering the aggressiveness of MES GBM, there is an urgent need to develop an optimal therapeutic strategy to treat devastating tumors.

Nuclear receptor (NR) PPARγ is expressed in brain cells, including astrocytes, microglia, oligodendrocytes, and neurons, where it controls cell growth and differentiation^10^. Several preclinical studies have reported beneficial effects of PPARγ agonizts against glioma growth^11,12^. Consistently, clinical studies reported subsets of patients presenting manifest therapeutic benefits of the drug, although overall analysis showed no statistical significance, which could be due to the small number of patients in the studies^13,14^. This suggests that PPARγ should be considered a prospective target for GBM therapy, and the data simultaneously raise an important set of issues regarding which subsets of individuals or specific tumor subtypes are responsive to PPARγ agonizts and what still needs to be elucidated regarding the molecular understanding of receptor action.

Here, we identified PPARγ as uniquely expressed in MES but not in PN GSCs by analyzing NR expression in an RNA-sequencing dataset. Further analysis using patient samples and public databases revealed the clinical association of the receptor with aggressive MES GBM as a potential pathologic diagnostic and prognostic biomarker. Biologically, PPARγ plays an important role in suppressing stemness as well as tumor growth of MES GSCs. In addition, the in vitro tumor suppressive function of PPARγ was confirmed using orthotopic and heterotopic xenograft mouse models.

Taken together, these results provide insight into PPARγ activity and provide a rationale for targeting PPARγ in aggressive MES GBM.

## Materials and methods

### Cell culture and reagents

GSCs, 448T, X01, X02, and 528 for PN and 0502, 83, and 1123 for MES, were cultured in DMEM/F-12 supplemented with B27 (Invitrogen, Carlsbad, CA), EGF (10 ng/ml, R&D Systems, Minneapolis, MN), bFGF (5 ng/ml, R&D Systems), 50 U/mL penicillin, and 50 U/mL streptomycin at 37 °C with 5% CO_2_. The cells were kindly provided by Jong Bae Park (National Cancer Center, South Korea) (448T, X01, X02, 528, 83, and 1123) and Myung-Jin Park (KIRAM, South Korea) (0502). Pioglitazone was purchased from Santa Cruz or Sigma-Aldrich. Troglitazone, SU6656, dasatinib, and gefitinib were obtained from Santa Cruz (Dallas, TX). 15-Deoxy-∆-11,13-prostaglandin J2 (15d-PGJ_2_), T0070907, azacitidine and bafilomycin A1 were from Sigma-Aldrich (St. Louis, MO). U0126 and helenalin were from Merck (Darmstadt, Germany) and ChemFaces (Hubei, China), respectively. ActiveMax^®^ Recombinant human TNF-alpha was obtained from Acrobiosystems (Newark, DE).

### Transcriptome sequencing

Total RNA concentration was measured by Quant-IT RiboGreen (Invitrogen). To assess the integrity of total RNA, samples were run on a TapeStation RNA ScreenTape instrument (Agilent). High-quality RNA samples showing RNA integrity numbers higher than 7.0 were chosen to construct an RNA library in which 1 µg of total RNA from each sample was used with a Illumina TruSeq mRNA Sample Prep kit (Illumina, Inc., San Diego, CA, USA). The first step in the workflow involved purifying poly-A-containing mRNA molecules using poly-T-attached magnetic beads. Following purification, mRNA was fragmented into small pieces using divalent cations under elevated temperature. The cleaved RNA fragments were copied into first strand cDNA using SuperScript II reverse transcriptase (Invitrogen) and random primers, which was followed by second strand cDNA synthesis using DNA Polymerase I and RNase H. These cDNA fragments then went through an end repair process, the addition of a single “A” base, and then ligation of the indexing adapters. The products were then purified and amplified by polymerase chain reaction (PCR) to create the final cDNA library. Libraries were quantified using quantitative PCR (qPCR) according to qPCR Quantification Protocol Guide (KAPA Library Quantification kits for Illumina Sequencing platforms) and were qualitatively analyzed using TapeStation D1000 ScreenTape (Agilent Technologies, Waldbronn, Germany). Indexed libraries were then sequenced using the HiSeq2500 platform (Illumina, San Diego, USA).

### Relative RT-PCR analysis

Total RNA was extracted using TRIzol reagent (Invitrogen) following the manufacturer’s instructions. RNA was then reverse-transcribed to generate cDNA using a qPCR RT Master Mix (Toyobo, Osaka, Japan). Real-time PCR (RT-PCR) was conducted with an ABI Prism 7900 HT Sequence Detection System (Applied Biosystems). Triplicates of each PCR were performed using SYBR green RT-PCR master mixes (Life Technologies). The delta delta Ct method was used to analyze 18 s as the reference gene. The primer sequences can be found in Table S3.

### Immunoblot assay

Cells or homogenized tissues were prepared in RIPA buffer, which was followed by immunoblot analysis as previously reported^15^. The following primary antibodies were used: β-actin (Abcam Cat# ab6276, RRID:AB_2223210), cyclin B1 (Santa Cruz Biotechnology Cat# sc-245, RRID:AB_627338), cyclin A (Santa Cruz Biotechnology Cat# sc-239, RRID:AB_627334), pStat3 (Y705) (Cell Signaling Technology Cat# 9131, RRID:AB_331586), Stat3 (Cell Signaling Technology Cat# 9139, RRID:AB_331757), SOX2 (Cell Signaling Technology Cat# 3728, RRID:AB_2194037), CD44 (Cell Signaling Technology Cat# 3570, RRID:AB_2076465), PPARγ (Cell Signaling Technology Cat# 2435, RRID:AB_2166051), Cyclin A2 (Cell Signaling Technology Cat# 4656, RRID:AB_2071958), p21 (Cell Signaling Technology Cat# 2947, RRID:AB_823586), pSrc (Cell Signaling Technology Cat# 6943, RRID:AB_10013641), pEGFR Y1773 (Cell Signaling Technology Cat# 4407, RRID:AB_331795), and LC3 (Cell Signaling Technology Cat# 4108, RRID:AB_2137703). For secondary antibodies, horse radish peroxidase (HRP)-conjugated anti-mouse IgG (Abcam Cat# ab6728, RRID:AB_955440) from Abcam and anti-rabbit IgG (Innovative Research Cat# G-21234, RRID:AB_1500696) from Invitrogen were used.

### Gene-expression analysis

Expression analysis of genes of interest was performed with a microarray dataset obtained from the GEO database (GSE67089) or from The Cancer Genome Atlas (TCGA) data obtained from the Nature dataset of cBioPortal^16–19^. Pearson correlation analysis was performed to determine the correlation between PPARγ and PN or MES markers.

### Survival analysis

Prognostic analysis was performed using datasets downloaded from a database from TCGA obtained from cBioPortal or from a REMBRANDT database from betastasis.com. A Kaplan–Meier plot was used to show patient survival on PPARγ and COUP-TFI expression or between PN and MES subtypes with a log-rank test performed for statistical significance analysis.

### MTS assay

MTS solution was prepared according to the manufacturer’s instructions (Promega, Madison, WI). At the end of the experiments, cell viability was determined by incubating cells with MTS solution for 2 h following OD measurement at 490 nm.

### In vitro limiting dilution assay and sphere forming assay

For the in vitro limiting dilution assay, decreasing numbers of GSCs (100, 80, 60, 40, 20, and 10) per well were seeded in 96-well plates containing media with DMSO or pioglitazone at a final concentration of 10 μM. Fourteen days later, the number of wells containing spheres with diameters > 100 μm was counted using an inverted microscope. Stem cell frequency was analyzed using software available at http://bioinf.wehi.edu.au/software/elda/.

Sphere formation assays were carried out in 96-well plates with 100 cells per well for fourteen days of treatment or 5000 cells per well for 3 days of treatment. The number of spheres with diameters > 100 μm was counted using an inverted microscope.

### Mitochondrial oxygen consumption rate

MES GSCs plated at 10^5^ cells/well in 6-well plates were treated with 10 μM pioglitazone for 48 h. Cells were then transferred to a Seahorse microplate (Agilent) precoated with BD Cell-Tak^TM^ (BD Biosciences, San Jose, CA) following the manufacturer’s instructions. Mitochondrial stress tests were performed as recommended by the manufacturer (Agilent). The basal oxygen consumption rate was measured, and then it was measured again after the following drug injections: 2 μM oligomycin, 0.5 μM FCCP, and 0.5 μM rotenone/antimycin A. The reads were normalized by total protein amounts measured with a Pierce BCA Protein Assay kit (Thermo).

### PPARγ knockdown using siRNA

For siRNA transfection, MES 0502 and 1123 GSCs were seeded at 5 × 10^4^ cells followed by reserve transfection siRNA control or the combination of three siRNAs targeting PPARγ at a final concentration of 100 nM each for 4 days with DharmaFECT 1 (Dharmacon) as the transfection reagent. The cell suspension was then collected for immunoblot assay and MTS assay to check knockdown efficiency and cell growth, respectively. The siRNA sequences can be found in Table S4.

### Adenovirus generation

Adenoviruses expressing BLRP-PPARγ (pAd-PPARγ) or adenovirus control (pAd-Dest) were generated using the ViraPower^™^ Adenoviral Expression System (Thermo) according to the manufacturer’s instructions. Briefly, BLRP-PPARγ, which was in a TOPO vector, was transferred to pAd-Dest vectors and then purified using phenol:chloroform:isoamyl alcohol 25:24:1 (Sigma-Aldrich). After digestion with PacI, plasmids were transfected into HEK293A cells until a cytopathic effect was observed. Crude viruses were then harvested and amplified in HEK293A cells for further experiments.

### Heterotopic xenograft tumor models

Animal experiments were approved by the Institutional Animal Care and Use Committee (IACUC) of Yonsei University Wonju College of Medicine (Approval number: YWC-170907-3). A xenograft model was established by subcutaneously injecting five million MES 83 cells into the right flank region of 4-week-old female Balb/c nude mice. Mice were randomly divided into two groups (the vehicle group *n* = 4 and the pioglitazone group *n* = 5) after tumors were tangible. Vehicle or pioglitazone (100 mg/kg) treatment was intraperitoneally administered every other day for 31 days. Tumor volume and body weight were measured every 2 days, and tumor weight was measured at the end of the experiment. Tumor volume was calculated using the formula ½ × (width^2^ × length) after measuring the tumor with a digital caliper.

### Orthotopic xenograft tumor models

Animal experiments were conducted in accordance with protocols approved by the Institutional Animal Care and Use Committee at the Korea Institute of Radiological & Medical Sciences, Republic of Korea. Totally, 10,000 GSCs (cell line 83) resuspended in 3 μl of DMEM/F12 medium were stereotactically transplanted into the left striatum of the brains of 5-week-old female BALB/c nude mice. The injection coordinates were 2.2 mm to the left of the midline and 0.2 mm posterior to the bregma at a depth of 3.5 mm. Mice were euthanized using CO_2_ when they showed severe weight loss with neurologic symptoms, which occurred approximately 8 weeks after xenograft transplantation. Mouse brains were harvested and fixed with 4% paraformaldehyde for immunohistochemistry (IHC) staining. The survival rate of mice was analyzed at the end of the experiment.

### Patient sample collection and preparation

Fresh tissue samples or ready-to-use paraffin embedded sections were obtained from Wonju Severance Christian Hospital under the approval of the Committee of Institutional Review Board (Approval number: CR318068). Fresh tumors were fixed with 4% paraformaldehyde for 48 h at 4 °C, and then they were placed in 30% sucrose at 4 °C until the tumors sank. After cryosectioning, samples were stained for proteins of interest.

### IHC staining

For observation of the histologic features, mouse brains fixed in paraformaldehyde were embedded in paraffin, sliced (10 μm thickness), and stained with hematoxylin and eosin (Merck Millipore). IHC staining was performed with frozen sections of fresh tissue samples or paraffin embedded tissues as described in the literature^20^. For paraffin samples, sections were rehydrated followed by antigen retrieval step by boiling samples in citrate buffer (pH 6.0) before continuing with other steps. Both frozen and paraffin sections were then blocked using DAKO Peroxidase Blocking Solution (Agilent) for 30 min. After washing with water, samples were permeabilized by incubation with PBST (PBS with 0.25% Triton X) for 30 min and then blocked in normal goat serum in PBST (1:100) for 30 min. Tissue sections were then incubated with primary antibodies overnight at 4 °C in a humidified chamber. After washing in PBST, samples were incubated in biotinylated universal antibody (horse anti-mouse/rabbit IgG) and ABC solution in a VECTASTAIN Elite ABC HRP kit (Vector, Burlingame, CA) following the manufacturer’s instructions. Samples were developed using ImmPACT DAB Peroxidase (HRP) Substrate (Vector) and were mounted with Permanent Mounting Medium (Vector).

For IHC staining of Ki67 in brain tumor xenograft sections, after the antigen retrieval process with citrate buffer (pH 6.0) and endogenous peroxidase blocking with 3% hydrogen peroxide, tissue sections were incubated in 1% bovine serum albumin blocking solution (v/v) for 0.5 h at room temperature and then with the primary antibody overnight at 4 °C in a humidified chamber. Bound antibody was detected by a Vectastain ABC kit (Vector) following the manufacturer’s instructions.

### Statistical analysis

All graphing and statistical analyses, including two-tailed Student’s *t* test, ANOVA, Pearson correlation coefficient and log-rank test, were performed using GraphPad Prism version 6.0 or 7.0. Data are presented as the mean ± SEM (*n* ≥ 3).

## Results

### PPARγ is a pathologic diagnosis marker for MES GBM

**Fig. 1:**
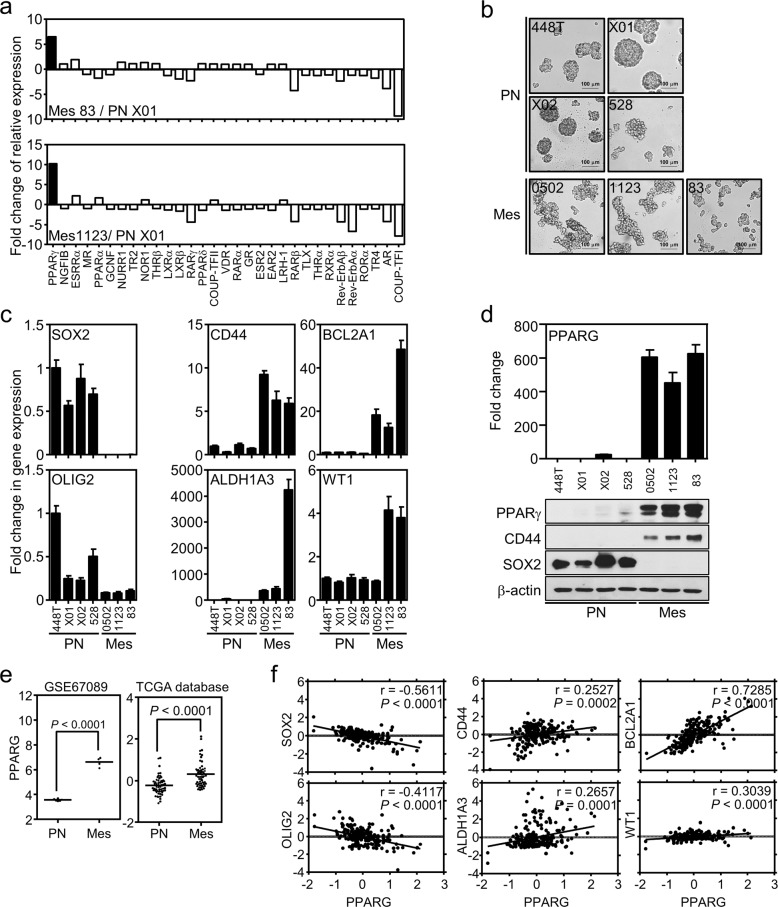
PPARγ is a diagnostic biomarker for MES GBM. **a** Fold difference in NR expression between MES- and PN-type GBM cells. RNA-seq analysis was performed for two MES (83 and 1123) and one PN (X01) type of GSC. **b**, **c** Characterization of GSCs. **b** Morphological features of four PN types (448T, X01, X02, and 528) and three MES types (0502, 1123, and 83) of GSCs. **c** Relative mRNA expression of biomarkers representing PN- and MES-type GBM in the panel of cell lines. A relative RT-PCR assay was carried out to measure the mRNA expression of biomarkers for PN (SOX2 and OLIG2) and MES (CD44, BCL2A1, ALDH1A3, and WT1) in the panel. The graph shows the mean ± S.E.M. (*n* = 3). **d** Both mRNA and protein expression of PPARγ are shown along with representative biomarkers in the GSC panel. The graph shows the mean ± S.E.M. (*n* = 3). **e** Identification of MES-featured PPARγ expression using public GBM datasets. PPARγ expression was analyzed in PN and MES types using public GEO (left) of TCGA databases (right). Each dot in the box represents individual GSC samples (left, *n* = 10) or tumor tissues (right, *n* = 112). The line indicates median expression in each group. **f** Pearson correlation of PPARγ expression to GBM biomarkers. PPARγ expression is positively correlated with MES markers (CD44, BCL2A1, ALDH1A3, and WT1) but negatively correlated with PN markers (SOX2 and OLIG2) when analyzed in the database from TCGA. Note that *r* and *P* represent the Pearson correlation coefficient and statistical significance, respectively.

Since multiple NRs are known to be involved in brain tumor pathogenesis^15,21,22^, we wondered which subsets of NRs are associated with GBM subtypes, and we focused specifically on PN and MES tumors. To this end, we first carried out RNA sequencing and analyzed the relative expression of 31 NRs in PN GSCs, MES GSCs, normal human astrocytes (NHAs), and normal neural stem cells (NSCs). We found 23 out of 31 NRs to exhibit no or marginal difference in expression between the two subtypes of GSCs, while 7 NRs showed distinct subtype-dependent expression patterns GSCs. Among those that were different were six NRs in the androgen receptor family, chicken ovalbumin upstream promoter-transcription factor I (CoupTF-I) I, retinoic acid receptor (RAR) beta, RAR gamma, reverse-erb (Rev-erb) alpha, and rev-erb beta for PN specific expression pattern, while PPARγ, interestingly, showed distinct expression that was five to tenfold higher in MES GSCs than it was in PN GSCs (Fig. 1a). Note that PPARγ expression is higher in MES GSCs, while Coup-TFI expression is higher in PN GSCs relative to that of NHAs as well as NSCs (Fig. S1a). The PPARγ expression from the RNA-seq analysis was validated at the mRNA and protein levels using subsets of PN and MES GSCs, of which cellular characteristics were confirmed by distinct morphological features as well as subtype-specific marker expression, as previously reported (Fig. 1b–d)^7^. PN markers used were SOX2 and Olig2, while MES markers used were CD44, ALDH1A3, WT1, and BCL2A1 (Fig. 1c). To verify the in vitro data of the GSC panel, we performed gene-expression analysis using public microarray datasets obtained from GEO (GSE67089) or TCGA databases obtained from the cBioPortal dataset^16–19^. Consistently, two independent datasets confirmed that PPARγ exhibited significantly higher expression in MES GSCs than in the PN subtype of GBM (Fig. 1e). Further biostatistical analysis showed a significant negative Pearson correlation of PPARγ with PN markers but a positive correlation with MES markers (Fig. 1f). Note that Coup-TFI expression is opposite to that of PPARγ expression but has no prognostic value in patient survival analysis (Fig. S1b). Taken together, these results suggest PPARγ as a potential pathologic diagnostic biomarker along with other previously known marker proteins for MES GSCs.

### Functional evaluation of endogenous PPARγ in MES GSCs

**Fig. 2:**
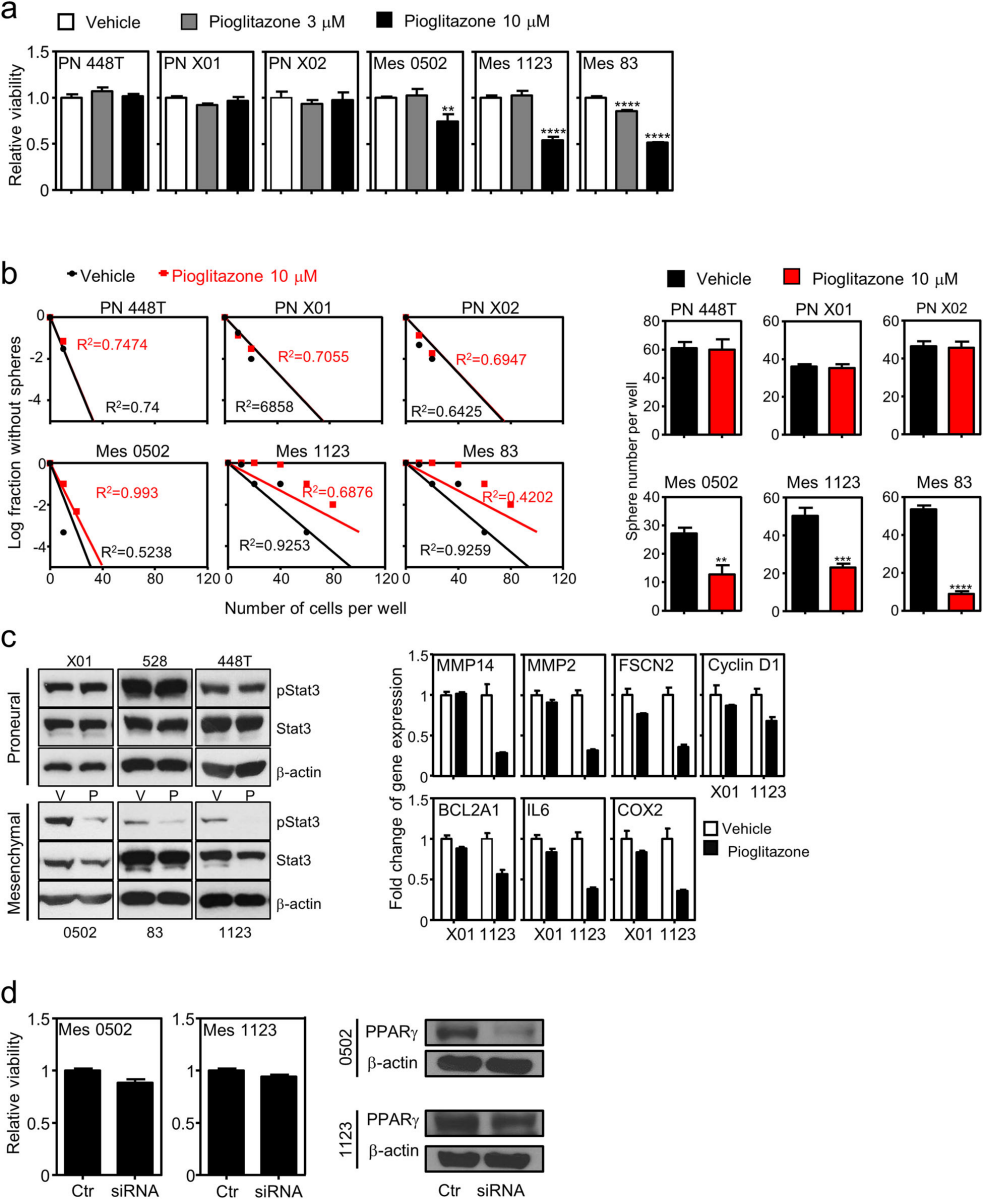
PPARγ activation suppresses tumor growth and stemness of MES GBM. **a** In vitro cell viability assay following pioglitazone treatment. PN or MES GSCs were treated with 3 or 10 μM pioglitazone for 7 days, which was followed by MTS assays of cell viability. Values are the mean ± SEM (*n* = 3). Asterisks are references as follows: **P < 0.01, and ****P < 0.0001 (one-way ANOVA, Tukey’s post hoc test). **b** PPARγ suppression of MES GSC stemness. Stemness was assessed by limiting dilution assays (left) and sphere forming assays (right) following pioglitazone treatment for 14 days. Stem cell frequency was calculated as described in the methods. Values are the mean ± SEM (*n* = 3). Asterisks are references as follows: **P < 0.01, ***P < 0.001, and ****P < 0.0001 (Student’s *t* test). **c** STAT3 signaling responsive to PPARγ activa*t*ion in MES but not in PN GSCs. PN and MES GSCs were treated with 10 μM pioglitazone for 48 h, which was followed by measuring the expression of pSTAT3 and target genes. Values are the mean ± SEM (*n* = 3). **d** In vitro cell viability assay following PPARγ knockdown. MES GSCs were transfected with a PPARγ siRNA, and after 4 days MTS assays (left) or immunoblot assays (right) were performed to assess knockdown efficiency. Values are the mean ± SEM (*n* = 3).

As we identified a unique expression pattern of PPARγ in MES GBM, we next wondered whether functional activation of the endogenous receptor provides any therapeutic benefits for treating the GBM subtype. Using GSC panels, we carried out experiments to measure cell viability and stemness upon PPARγ ligand treatment using MTS, limited dilution and sphere forming assays. Cell viability significantly decreased following treatment with synthetic agonizts, pioglitazone and troglitazone, for 7 days in PPARγ-positive MES GSCs but not in PPARγ-negative PN GSCs (Figs. 2a and S2a). Note that a well-known endogenous ligand of PPARγ 15d-PGJ_2_ did not affect cell viability (Fig. S2b), while unexpectedly, the PPARγ antagonist T0070907 reduced the cell viability of MES GBM (Fig. S2c). Moreover, stem cell frequency and sphere forming ability were notably reduced in MES but not PN GSCs under the same pioglitazone treatment conditions (Fig. 2b, Table S1, and Fig. S2d). Since STAT3 is known as a master regulator of MES transformation and glioblastoma stemness^23,24^, we examined STAT3 signaling in GSCs under pioglitazone treatment. We found that basal activation of STAT3 is significantly higher in PN than it is in MES GSCs. However, interestingly, the inhibition of STAT3 phosphorylation and the expression of its target gene occurs only in MES but not PN GSCs following pioglitazone treatment (Fig. 2c), suggesting PPARγ activation-dependent suppression of STAT3 signaling in MES GSCs. This is consistent with previous reports in which TZD treatment suppresses STAT3 phosphorylation to reduce inflammation^25,26^. We next examined the biochemical function of receptor activation to determine whether STAT3 suppression is associated with mitochondrial function in MES GSCs. However, MES GSCs showed no change in mitochondrial stress upon ligand activation of the endogenous receptor (Fig. S2e). Further loss-of-function analysis revealed that knocking down the receptor results in no cell growth inhibition of MES GSCs, indicating that endogenous PPARγ may be functionally inactive in MES GSCs (Fig. 2d). Taken together, these data suggest that the therapeutic potential of PPARγ can be exploited specifically for decreasing MES GSC progression.

### Gain of PPARγ function suppresses tumorigenicity of PN GSCs

**Fig. 3:**
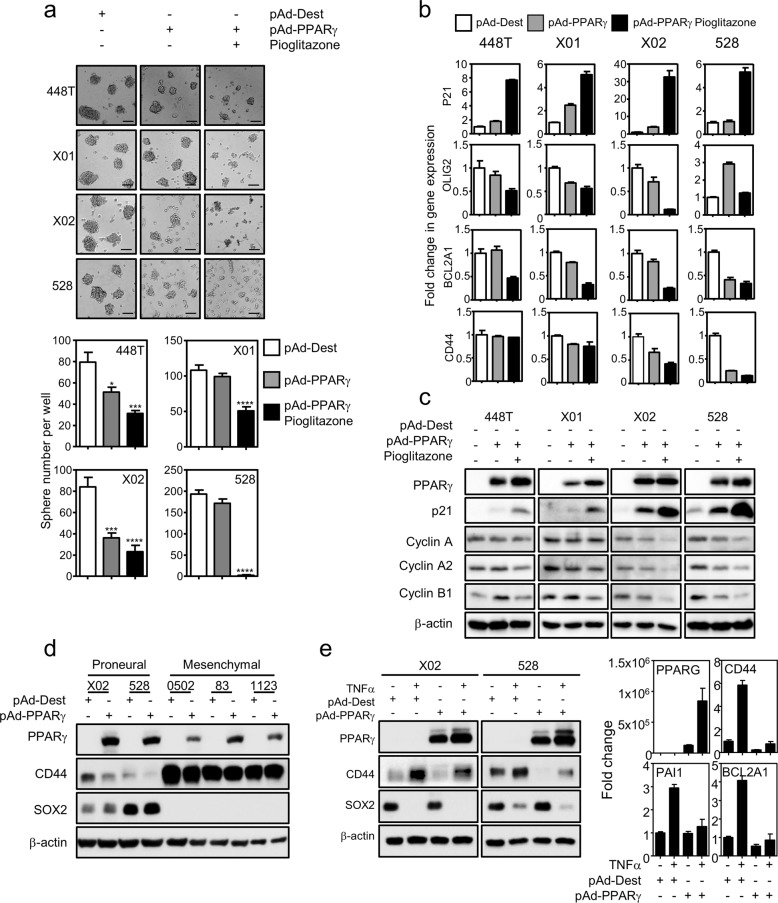
Exogenous expression of PPARγ inhibits the PMT process. **a** Exogenous overexpression of PPARγ suppresses sphere formation of PN GSCs. PN GSCs were infected overnight with adenovirus expressing control vector (pAd-Dest) or PPARγ (pAd-PPARγ) followed by 10 μM pioglitazone treatment for 3 days. Sphere forming ability (upper) or quantification of sphere number (lower) is represented. Data represent the mean ± S.E.M. (*n* = 5). Asterisks are references as follows: ***P < 0.001, and ****P < 0.0001 (one-way ANOVA, Tukey’s post hoc test). **b**, **c** Gene expression for GBM biomarkers or cell cycle regulation. **b** mRNA expression of p21, Olig2, Bcl2a1, and CD44. **c** Immunoblot assay for cell cycle proteins. PN GSCs were infected overnight with adenovirus expressing control vector (pAd-Dest) or PPARγ (pAd-PPARγ), which was followed by 10 μM pioglitazone treatment for 24 h. Data represent the mean ± S.E.M. (*n* = 3). **d** Immunoblot analysis of CD44 and SOX2 in PN and MES GSCs infected with pAd-Dest control or pAd-PPARγ for 2 days. **e** PMT assay upon PPARγ overexpression. Expression of PN and MES markers was analyzed in PN cells infected with pAd-Dest control or pAd-PPARγ with/without TNFα treatment. PN GSCs were treated daily with 50 ng/ml TNFα for 4 days in the presence of adenovirus expressing pAd-Dest control or pAd-PPARγ. Immunoblot assay of PPARγ, CD44, and SOX2 (left) or qPCR analysis of PPARγ and multiple MES markers, CD44, PAI1, and BCL2A1. Data represent the mean ± S.E.M.

As activation of endogenous PPARγ suppresses the growth and stemness of MES GSCs, we next performed a gain of functional approach for PPARγ in PN GSCs that exhibit no expression of endogenous PPARγ. Overexpression of PPARγ decreased the stemness of PN GSCs by increasing the adherence of the cells, which became more prominent under pioglitazone treatment (Fig. 3a). This was molecularly confirmed by examining decreased expression of the stemness gene OLIG2, the antiapoptotic gene BCL2A1 or the MES gene CD44. Note that the expression of PPARγ and its target genes, FABP4, PDK4, and LPL, was confirmed upon PPARγ overexpression by quantitative RT-PCR^27–29^ (Fig. S3a). In addition, PPARγ overexpression decreased PN GSC proliferation by regulating cell cycle proteins; there was increased p21 expression and decreased expression of cyclin A, cyclin A2, and cyclin B1 (Figs. S3b and 3b, c). In addition, we surprisingly noticed that both mRNA and protein expression of CD44, an MES marker, was reduced upon PPARγ overexpression in PN GSCs (Figs. 3b, d and S3c). As some recent studies have suggested that the PMT process following radiation or chemotherapy treatment contributes to the development of increasingly aggressive GBM^8,19,30^, we wondered whether PPARγ overexpression and activation could suppress the MES GBM phenotype. To answer this question, we established a TNFα-induced PMT assay using PN X02 and 528 cells, which have been previously reported^8^, and then we tested the effect of PPARγ overexpression. Note that TNFα-induced PMT was confirmed by SOX2 reduction and CD44 upregulation in the cells. The exogenous expression of PPARγ reduced TNFα-induced MES markers CD44, PAI1, and BCL2A1 (Figs. 3d, e and S3d). Collectively, these data support the tumor suppressive role of PPARγ in GBM by suppressing stemness and attenuating TNFα-induced PMT.

### In vivo therapeutic evaluation of PPARγ for MES GBM tumors

**Fig. 4:**
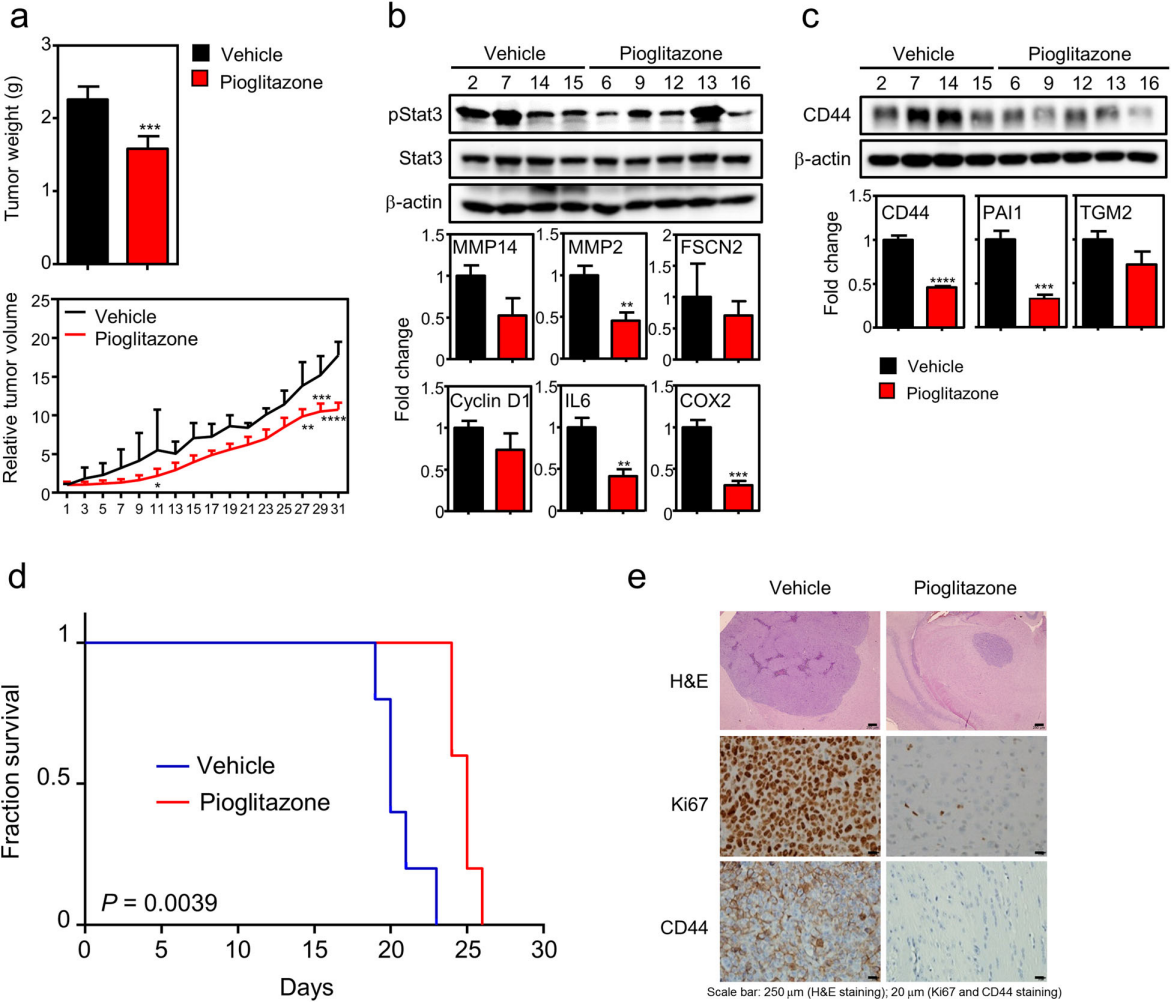
PPARγ is a therapeutic target for MES GBM. **a** In vivo analysis of xenograft tumors with pioglitazone treatment. Five millions of MES 83 GSCs were injected into the flank region of athymic nude mice. When tumors were visible, mice were intraperitoneally administered vehicle (*n* = 4) or 100 mg/kg pioglitazone (*n* = 5) every other day for 31 days. Both tumor weight (upper) and volume (lower) were measured every other day or at the end of the experiment, respectively. Tumor growth is represented as the relative mean tumor size ± SEM. Asterisks are references as follows: **P < 0.01, ***P < 0.001, and ****P < 0.0001 (Student’s *t* test (upper) and two-way ANOVA, Sidak’s post hoc test (lower)). **b**, **c** Gene-expression analysis in individual tumor samples upon pioglitazone treatment. Genes involved in STAT3 signaling (left) or MES markers (right) were assayed in the residual tumor tissues at the end of the in vivo experiment. **d** Survival analysis of the orthotopic mouse model. Orthotopic xenograft tumors were established by intracranial injection of one thousand 83 cells, followed by survival analysis. Kaplan–Meier plots are presented to show the survival of mice intracranially established with MES 83 GSCs with (*n* = 5) or without (*n* = 5) oral administration of 100 mg/kg pioglitazone for 3 weeks. A log-rank test was used for the statistical analysis. **e** H&E and IHC staining for Ki67 and CD44 expression in representative tumor sections from the orthotopic models.

Having demonstrated that PPARγ activation could suppress MES GSC growth as well as stemness, we further wanted to determine the therapeutic potential of NRs using an in vivo xenograft mouse model. For in vivo analysis, we established both orthotopic and heterotopic xenograft tumor models using the 83 MES GSCs. Heterotopic tumors subcutaneously established on the right flank of athymic nude mice were treated with 100 mg/kg pioglitazone or vehicle every other day for 31 days when tumor size became visible. Consistent with the in vitro results, pioglitazone treatment suppressed tumor growth with no accompanying change in body weight (Figs. 4a and S4a). Moreover, to consider drug delivery through the blood brain barrier as well as the tumor microenvironment effect, an orthotopic tumor model established by intracranial injection of the same MES GSCs was utilized for further survival analysis of mice upon pioglitazone treatment. Consistently, PPARγ activation by pioglitazone significantly increased mouse survival compared to that of the control mice without significantly changing the body weight (Figs. 4d and S4b). Further molecular analysis of residual orthotopic and heterotopic tumor tissues treated with the drug revealed decreased Ki67 levels and STAT3 signaling, as well as a decrease in MES phenotypes, which reveals the therapeutic potential of pioglitazone in vitro and in vivo (Fig. 4b, c, e). Taken together, these results suggest that endogenous PPARγ could be targeted to suppress MES GBM progression upon ligand activation.

### High expression of PPARγ in the MES type of GBM

**Fig. 5:**
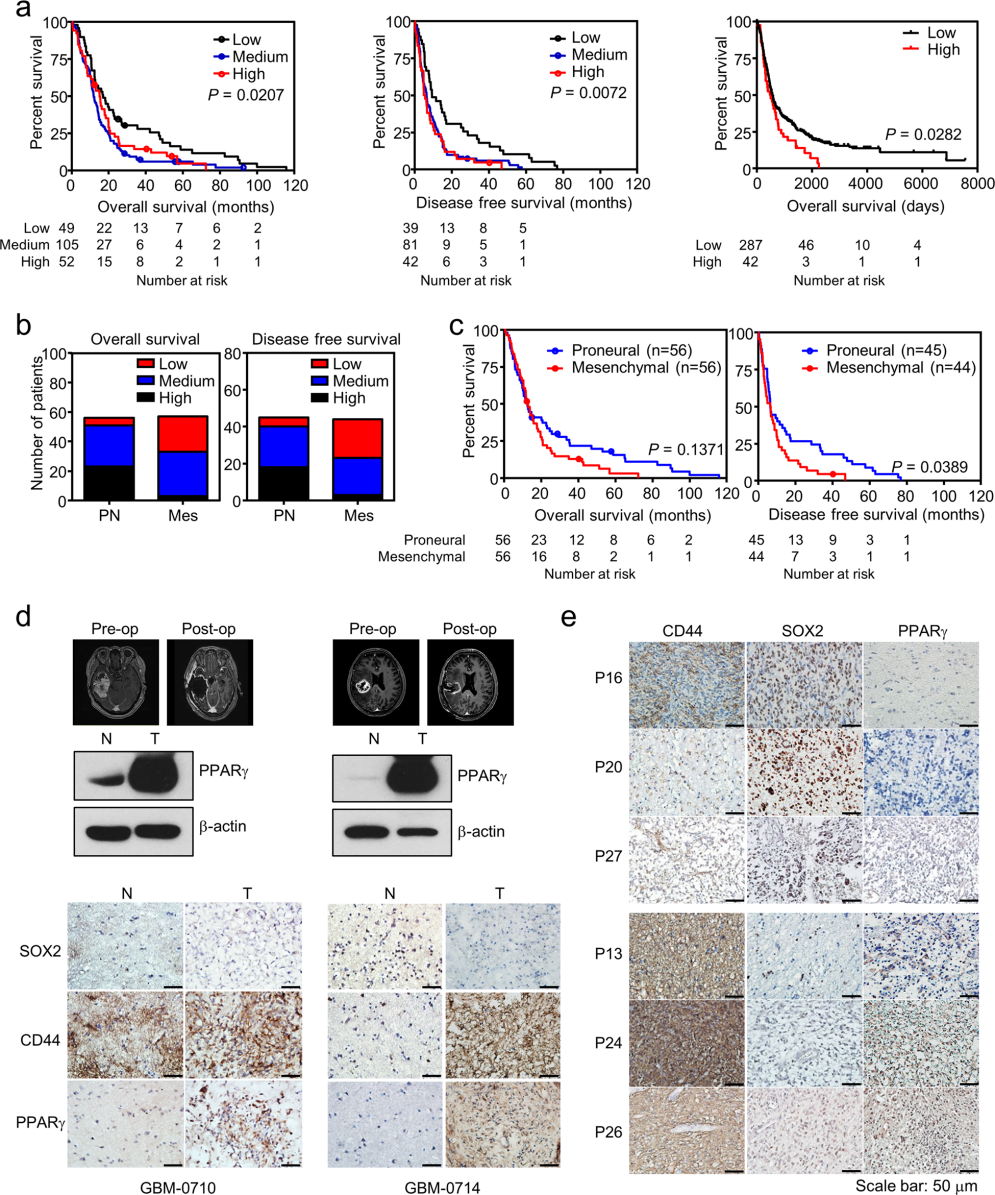
PPARγ is a potential theragnostic biomarker for GBM patients. **a** High PPARγ expression is associated with poor prognosis in GBM patients. Kaplan–Meier plots were generated for GBM patient survival upon PPARγ expression using a publicly available database. Overall survival (left, *n* = 206) and disease-free survival (middle, *n* = 162) were analyzed using data from the TCGA database or overall survival using the REMBRANDT database (right, *n* = 329) from http://www.betastasis.com/glioma/rembrandt/. **b** Distribution of PN and MES GBM patients based on PPARγ expression levels. All GBM patients in the database from TCGA used in (**a**) were counted based on low, medium, or high PPARγ expression levels. **c** Kaplan–Meier plots for overall survival (left) or disease-free survival (right) of PN and MES GBM patients in the same TCGA database. **d** PPARγ expression in microdissected GBM tissues. The microdissection process was carried out to dissect tumors from the corresponding normal tissues based on MRI images (upper), which was followed by an immunoblot assay for PPARγ (middle) and immunohistochemistry staining for PPARγ, SOX2, and CD44 (lower). Note that two pair-matched tissues were obtained from GBM patients. The scale bar represents 50 μm. **e** IHC of human high-grade glioma for PPARγ, SOX2, and CD44. The scale bar represents 50 μm.

In this study, we have demonstrated that upregulation of PPARγ expression specifically in MES GSCs may serve as a molecular signature representing pathologic diagnosis biomarkers as well as therapeutic targets for that particular tumor subtype. Along with preclinical results, we further wondered whether NR expression could provide any prognostic value. Herein, we performed biostatistics analysis using public datasets available from TCGA and REMBRANDT databases and interestingly found that high PPARγ expression is significantly associated with poor prognosis of GBM patients regarding both disease-free and overall survival (Fig. 5a). Further analysis of the receptor expression in GBM subtypes showed that the receptor expression level ranged from low to medium in PN GBM and from medium to high in MES GBM (Fig. 5b and Table S2). Consistent with this observation, the PN group showed a better prognosis of overall and disease-free survival than did the MES group (Fig. 5c). To confirm the results from public datasets, we examined PPARγ expression in GBM patient tissues, where two pairs of normal and corresponding tumor samples prospectively and six paraffin embedded tumor tissues retrospectively were obtained. PPARγ expression was dramatically upregulated in GBM tumors compared to the corresponding normal tissues in the two patients, and the tumor tissues showed CD44-positive expression, indicating an MES subtype (Fig. 5d). Note that one of the two patients had a recurrence of GBM at 1 year after surgery. Furthermore, PPARγ expression increased in recurrent tumors that acquired MES features with CD44 expression, while primary tumors expressed SOX2, indicating the PN subtype (Fig. S5a). Further retrospective analysis of tissue samples consistently showed that PPARγ expression was associated with MES GBM (Fig. 5e). In addition, it is important to note that PPARγ expression was not observed in brain meningioma (Fig. S5b). Collectively, these results suggest that PPARγ expression is a prognostic biomarker for MES GBM.

## Discussion

**Fig. 6:**
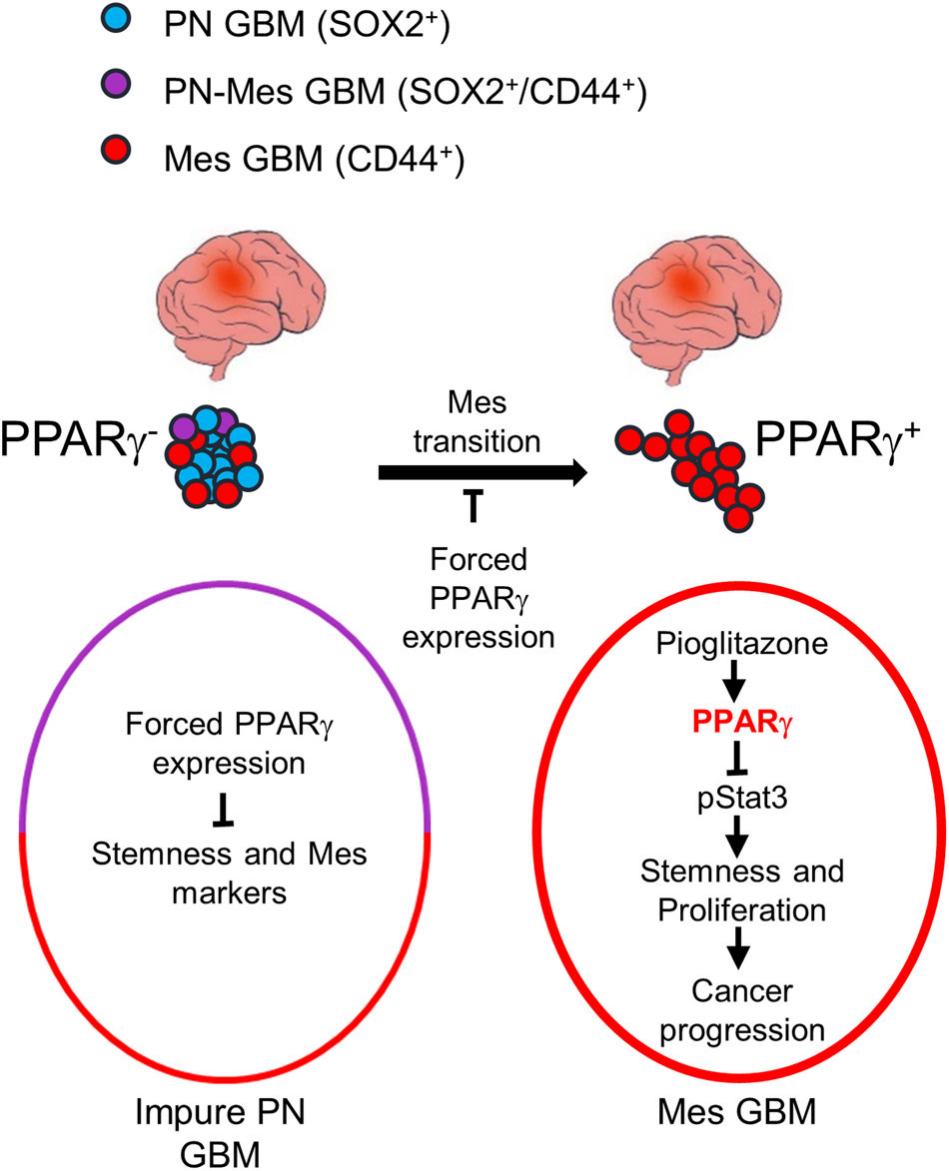
Hypothetical model of PPARγ as a therapeutic target in GBM. Among two mutually exclusive subtypes of GBM, PPARγ is specifically expressed in mesenchymal type. In proneural GBM, the overexpression of PPARγ reduced stemness and mesenchymal markers. The recovery of PPARγ in proneural type during proneural-mesenchymal transition attenuated this process. In mesenchymal GBM, the activation of endogeous PPARγ by ligands inhibited GBM progression.

GBM is one of the deadliest tumors in humans due to poor clinical outcomes resulting from therapeutic resistance, which leads to poor quality of life of patients. While clinical and molecular features have been characterized for tumor subtypes and a recent noticeable study identified the GBM origin initiating at the subventricular zone of the brain with aging, intratumoral heterogeneity, and cellular plasticity via PMT mainly contribute to tumor recurrence and have become a major hurdle in treating this devastating disease. Unlike other human tumors, therapeutic options for GBM patients are limited to standard radiation and chemotherapeutics including temozolomide, irinotecan, carboplatin, or bevacizumab after a postoperative resection; therefore, developing new therapeutic schemes or drug repositioning would greatly benefit patients suffering from fatal disease. Here, we systemically evaluated the therapeutic potential of PPARγ as a target for MES GBM treatment. To that end, we incorporated multiple independent, molecular and cellular approaches using in vitro as well as in vivo models. Furthermore, these preclinical results were clinically correlated with human GBM tissues and patient survival outcomes using public datasets. In brief, we found that (1) PPARγ expression is specifically upregulated in MES GSCs as well as tissues, and it is associated with poor prognosis of GBM patients; (2) biologically, PPARγ activation attenuates PMT and reduces stemness and viability of MES GSCs, which occurs mechanistically due to the suppression of the STAT3 signaling pathway; and (3) two in vivo xenograft tumor models independently confirmed the therapeutic potential of PPARγ upon pioglitazone treatment (Fig. 6). While we here proposed the clinical potential of PPARγ, it is important to have further discussion on the biological and clinical implications related to a couple of issues raised by this study. First, we have shown that high expression of PPARγ in GBM is associated with poor prognosis. It seems that PPARγ upregulation may promote malignant GBM development, but ligand-mediated activation of the receptor could have a tumor suppressive function. Indeed, further subtype analysis revealed that poorer prognosis in the Kaplan–Meier survival plot was associated with a higher number of MES subtypes of GBM patients. In addition, in vitro knockdown of the receptor in MES GSCs showed no growth disadvantage (Fig. 2d), which may further strengthen the idea of a function for endogenous PPARγ upon ligand activation. Consistent with this notion, we previously proposed that the antitumor effect of TZDs is mediated by PPARγ activation in lung cancer^31^. Other studies reported that TZDs not only eradicate quiescent leukemia stem cells, which are responsible for cancer recurrence, but also increase the anticancer effect of BCR-ABL1 inhibitor, one of the first-line therapies for leukemia treatment^32,33^. In considering patients with lung cancer harboring mutant EGFR or HER2-positive breast cancer (BC) patients, these cancers are generally known to show a worse prognosis than other subtypes of the same cancer. However, targeted therapeutics against these receptors reversely benefit cancer patients with the corresponding mutations^34,35^. Likewise, PPARγ upregulation would become paradoxically beneficial to MES GBM patients.

Second, one might ask what upstream factor is involved in regulating PPARγ expression and function during the PMT process. To answer this question, we assessed multiple candidate factors or pathways known to regulate endogenous receptor expression and activation. This included autophagy signaling^36^, epigenetic regulation of the receptor^37^, and transcriptional regulation by C/EBPβ ^38^ for PPARγ expression^38^, and Src, c-Abl, EGFR, and MEK kinases known to phosphorylate the receptor for activity regulation^39–43^. We found no evidence that any of these factors is involved in receptor expression or activation in MES GSCs (Fig. S6). Therefore, it is still important to identify upstream regulators for MES expression of PPARγ in GBM.

Third, interestingly, we observed that exogenous overexpression of PPARγ reduces the TNFα-induced PMT process. From the finding of decreased cell viability and stemness of MES GSCs upon PPARγ activation, a therapeutic strategy recovering receptor expression would be considered to prevent cellular plasticity of PN into the MES GSC subtype, which potentially attenuates intratumoral GBM heterogeneity. This is intuitive if PPARγ is considered an antistemness factor in general^44,45^ and would be further rationalized as a potential chemopreventive target against both PN and MES GBM incidence. In addition, it would be of interest to investigate whether PPARγ expression is induced in relapsed GBM following temozolomide or radiation therapy since, recurrent GBMs are generally known to acquire MES features.

Taken together, this study proposes that the nuclear receptor PPARγ has clinical potential as a pathological diagnostic and prognostic biomarker and a therapeutic target for MES GBM.

## Supplementary information

Supplementary information_Clean version
