# Supplementary material for: Peroxisome proliferator-activated receptor gamma as a theragnostic target for mesenchymal-type glioblastoma patients
Source: Exp Mol Med. 2020 Apr 13;52(4):629–42. doi: 10.1038/s12276-020-0413-1 (PMC7210935; doi:10.1038/s12276-020-0413-1)
Supplement: Supplementary file 1 — Supplementary information_Clean version [file 12276_2020_413_MOESM1_ESM.pdf]

Supplementary information

Peroxisome Proliferator-Activated Receptor gamma as a Theragnostic Target for Mesenchymal-type Glioblastoma Patients

Tuyen N.M. Hua, Jiwoong Oh, Sohyun Kim, Jayson M. Antonio, Vu T.A. Vo, Jiyeon Om, Jong-Whan Choi, Jeong-Yub Kim, Chan-Woong Jung, Myung-Jin Park*, and Yangsik Jeong*

The supplementary information contains 6 figures and 4 tables

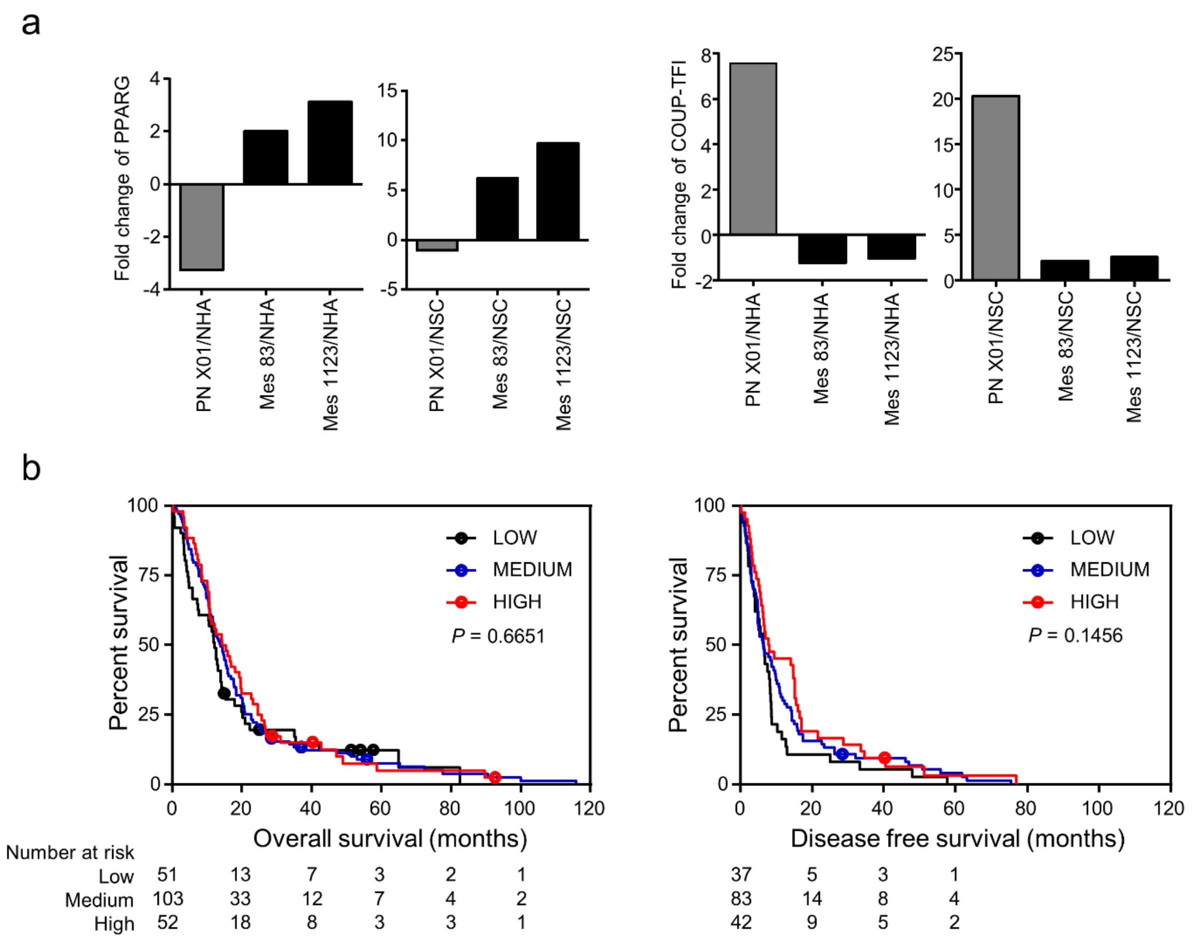

Fig. S1. Analysis of PPARγ and COUP-TFI expression in PN or MES GSCs versus normal human astrocyte (NHA) or normal neural stem cell (NSC). (a) High expression of PPARγ in MES GSCs (left) or COUP-TFI in PN GSCs (right) compared to NHA and NSC analyzed by RNA-seq. (b) Prognostic value of COUP-TFI in GBM. Kaplan-Meier plots were represented for survival of GBM patients upon COUP-TFI expression in public database. Overall survival (left, n=206) and disease free survival (middle, n=162) were analyzed using TCGA dataset.

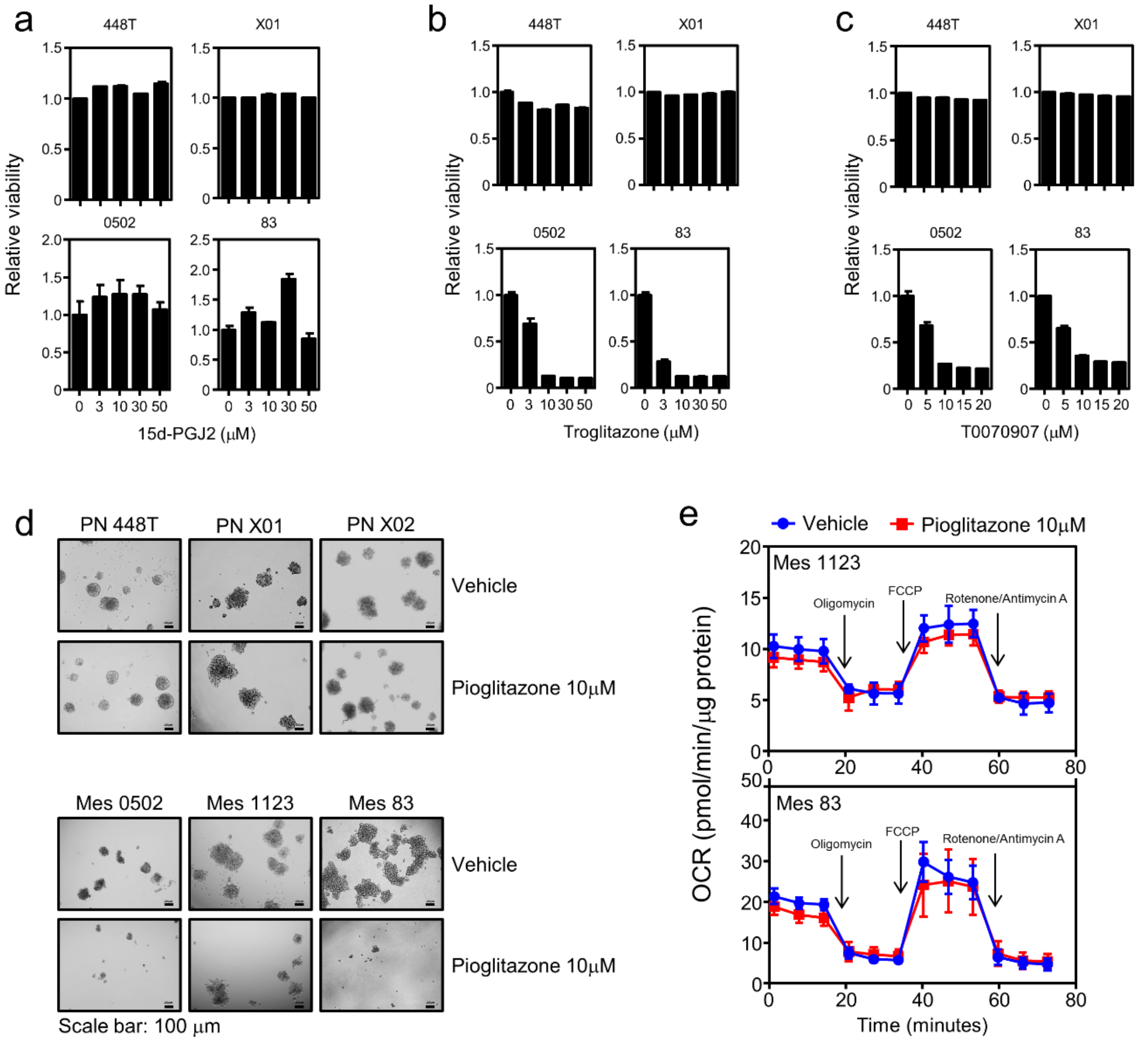

Fig. S2. *In vitro* cell viability assay upon multiple treatments. PN or MES GSCs were treated with 15d-PGJ2 (a), troglitazone (b) or T0070907 (c) in a dose dependent manner for 7 days and followed by MTS assay for cell viability analysis. Value are mean ± SEM (n=3). (d) Sphere forming capability upon pioglitazone treatment in GSCs. Photos of PN and MES GSCs upon 10 μM of pioglitazone treatment for 14 days. Scale bar represents 100 μm. (e) Oxygen consumption rate (OCR) in MES GSCs with pioglitazone treatment. Cells were treated with 10 μM of pioglitazone for 2 days followed by measuring OCR as described in method.

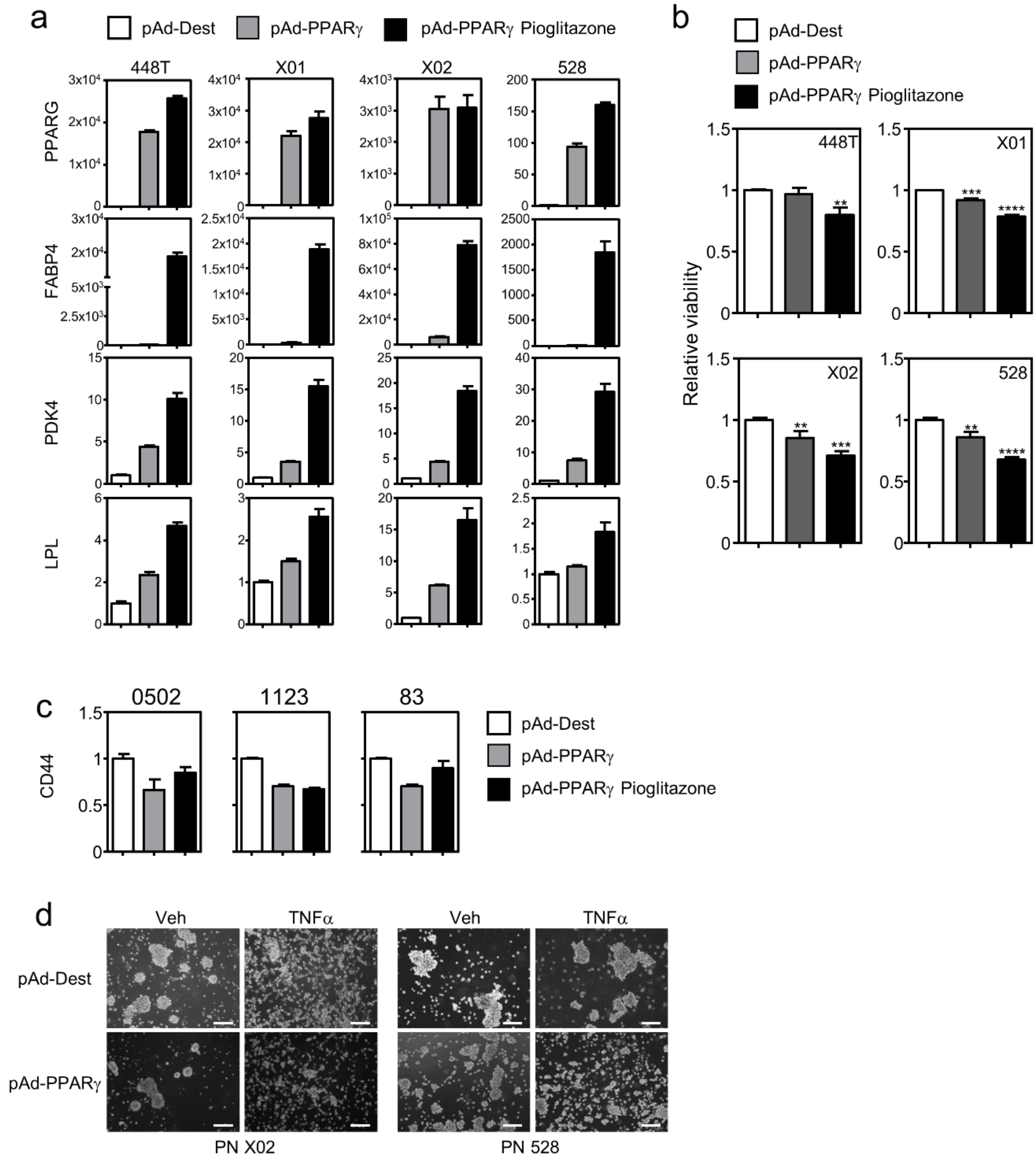

Fig. S3. Exogenous expression of PPARγ serves as a tumor suppressor in PN GSCs. (a) mRNA expression of PPARγ and target genes in PN GSCs. PN GSCs were transduced with pAd-Dest control or pAd-PPARγ overnight followed by 10 μM of pioglitazone treatment for 24 h. Data represent mean ± S.E.M. (n=3). (b) *In vitro* cell viability assay upon PPARγ overexpression and/or activation. PN GSCs were transduced with adenovirus harboring pAd-Dest control or pAd-PPARγ overnight followed by 10 μM of pioglitazone treatment for 3 days. Cell viability was assessed using MTS assay. Asterisks refer to ** $P < 0.01$, *** $P < 0.001$, **** $P < 0.0001$ (one-way ANOVA, Tukey's post-hoc test). (c) mRNA expression of CD44 in MES

GSCs infected with the corresponding adenoviruses. Cells were transduced overnight with adenoviruses with control or PPARγ expression plasmid and followed by pioglitazone 10 μM treatment for 24 h. Data represent mean ± S.E.M. (n=3). (D) Representative pictures of morphology of PN GSCs with pAd-Dest control or pAd-PPARγ in TNFα-induced PMT process. PN cells were daily treated with 50 ng/mL of TNFα for 4 days in the presence of adenovirus expression pAd-Dest control or pAd-PPARγ. Scale bar represent 500 μm.

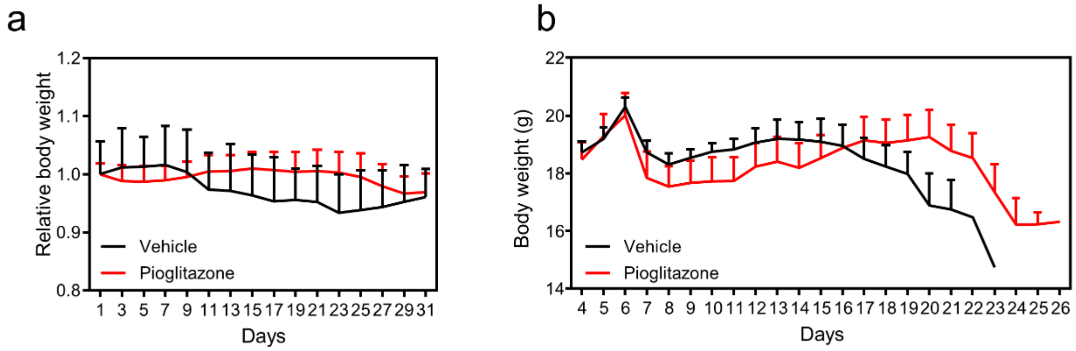

Fig. S4. Monitoring body weight change of the *in vivo* tumor models. (a) MES 83 GSCs were xenografted into the flank region of nude mice. Mice were intraperitoneally administered with vehicle (n=4) or pioglitazone 100 mg/kg (n=5) for 31 days. Body weights were measured every other day and relative body weights are shown as mean relative body weight ± SEM. (b) Body weight of mice intracranially injected with MES 83 GSCs with vehicle (n=5) or pioglitazone 100 mg/kg (n=5). Body weights were measured every day.

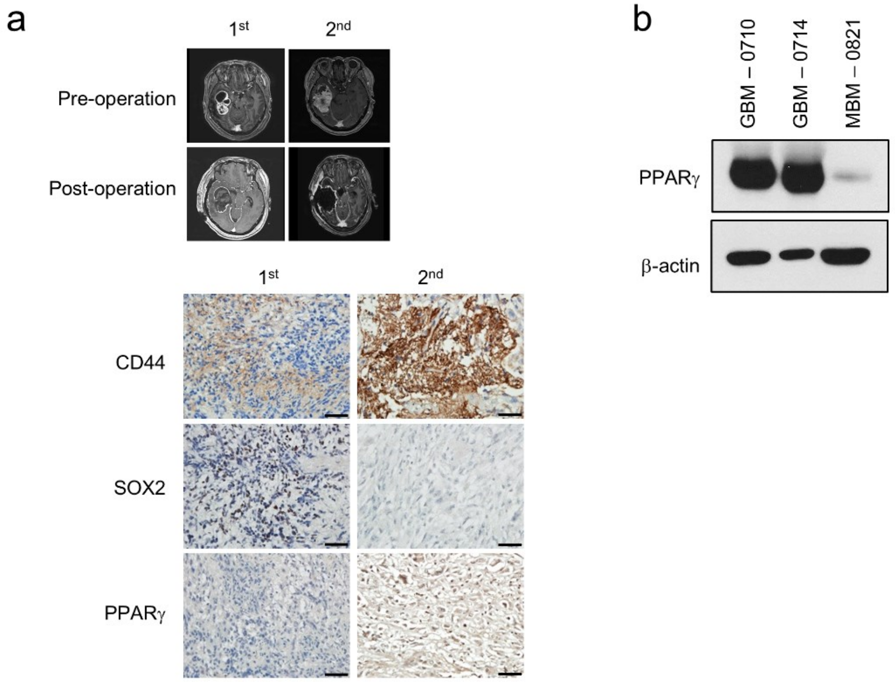

Fig. S5. PPARγ expression in GBM patients. (a) MRI images (upper) and immunohistochemistry for PPARγ, SOX2 and CD44 expression (lower) in primary (left) tumor and recurred tumor (right) from the same patient. (b) Immunoblot analysis for PPARγ expression in GBM or brain meningioma tissues. Scale bar: 50 μm.

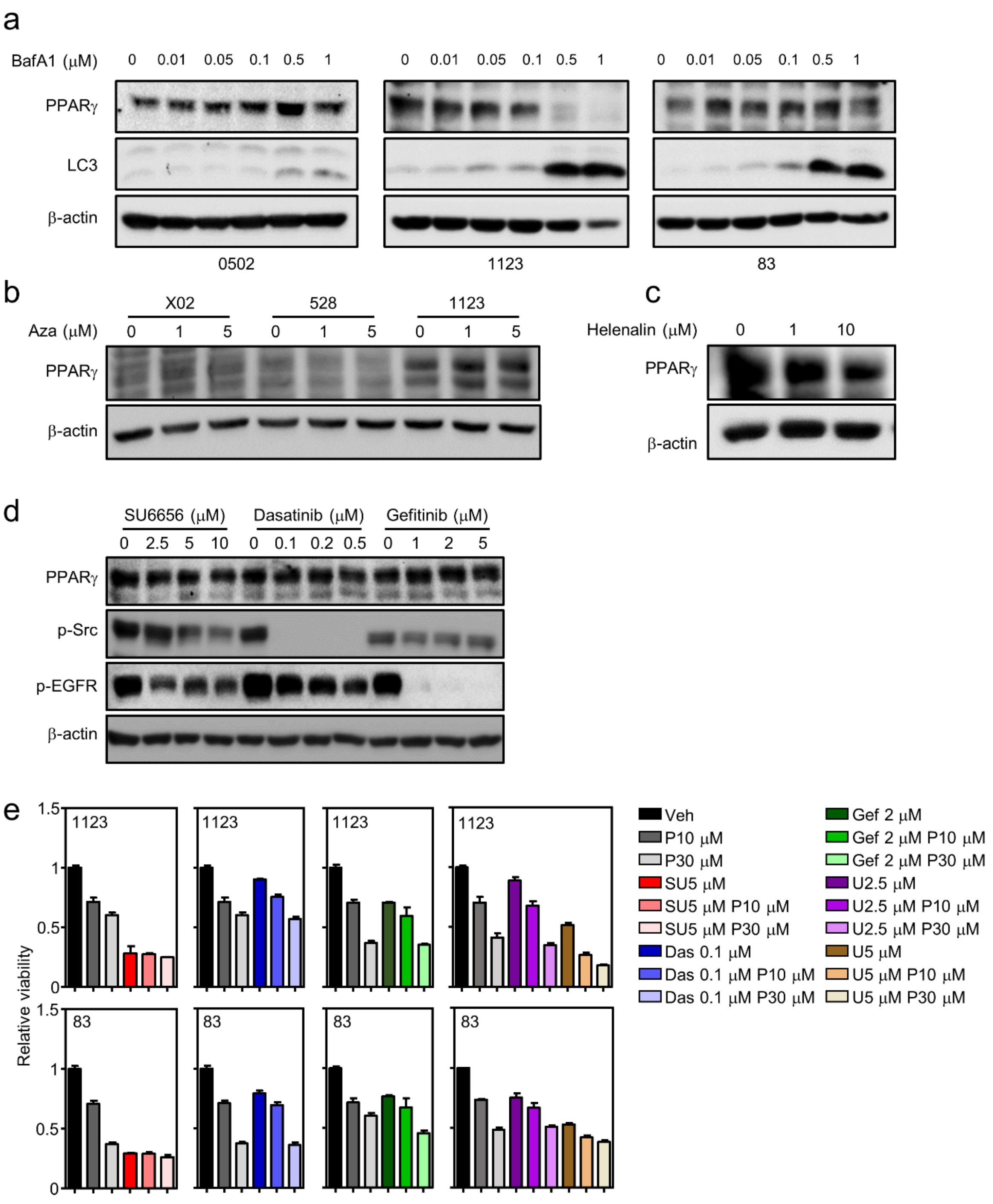

Fig. S6. Pharmacological assessment for potential upstream factors regulating PPARγ expression in GBMs. (a) MES GSCs were treated with autophagy inhibitor bafilomycin A1for 36 h followed by immunoblot for PPARγ and LC3 expressions. (b) PN and MES GSCs were treated with DNA methylation inhibitor Azacitidine for 72 h followed by immunoblot for PPARγ expression. (c) MES 1123 GSCs was treated with

C/EBPβ inhibitor helenalin in 48 h followed by immunoblot for PPARγ expression. (d) MES 83 GSCs were treated with Src inhibitor SU6656, dual Src and c-Abl inhibitor dasatinib or EGFR inhibitor gefitinib followed by immunoblot for proteins of interest. (e) Cell viability of MES GSCs upon pioglitazone (P) in combinations of multiple kinase inhibitors including Src inhibitor SU6656 (SU), dual Src and c-Abl inhibitor dasatinib (Das), EGFR inhibitor gefitinib (Gef) and MEK inhibitor U0126 (U) for 7 days. Data represent mean ± S.E.M.

Table S1. Stem cell frequency of PN or MES cells treated with pioglitazone

	1/(stem cell frequency)		*P* value
	Veh	Pioglitazone	
PN448T	7.92	9.52	0.476
PN X01	28.2	29.4	0.819
PN X02	25.3	28.4	0.54
MES 0502	4.6	12.5	0.000373
MES 1123	33.6	93.8	2.35E-07
MES 83	30.9	160.5	7.98E-15

Table S2. Subtype analysis of GBM patients in the TCGA datasets.

	Overall Survival			Disease Free Survival		
	Lower	Middle	Upper	Lower	Middle	Upper
**Total**	**49**	**105**	**52**	**39**	**81**	**42**
**Censored**	2	5	5	0	1	1
**PN**	1	1	1	0	0	0
**CL**	1	1	0	0	0	0
**N**	0	3	1	0	1	0
**MES**	0	0	3	0	0	1
**US**	0	0	0	0	0	0
**Event number**	47	100	47	39	80	41
**PN**	22	27	4	18	22	5
**CL**	17	34	1	14	28	1
**N**	1	9	15	1	9	12
**MES**	3	29	21	3	20	20
**US**	4	1	6	3	1	3

PN: proneural; CL: classical; N: neural; MES: mesenchymal; US: unknown subtype

Table S3. Primer sequences for RT-PCR

Gene name	Sequence (5'-3')
18S	Forward: ACCGCAGCTAGGAATAATGGA<br>Reverse: GCCTCAGTTCCGAAAACCA
SOX2	Forward: AACCCCAAGATGCACAACTC<br>Reverse: CGGGGCCGGTATTTATAATC
OLIG2	Forward: CTCCTCAAATCGCATCCAGA<br>Reverse: AGAAAAAGGTCATCGGGCTC
CD44	Forward: TACAGCATCTCTCGGACGGA<br>Reverse: CCCTTCTATGAACCCATACCTGC
BCL2A1	Forward: ATGGATAAGGCAAAACGGAG<br>Reverse: TGGAGTGTCCTTTCTGGTCA
ALDH1A3	Forward: TCTCGACAAAGCCCTGAAGT<br>Reverse: TATTCGGCCAAAGCGTATTC
WT1	Forward: TACACACGCACGGTGTCTTCA<br>Reverse: CTCAGATGCCGACCGTACAAG
PPARG	Forward: AGATCCAGTGGTTGCAGATTA<br>Reverse: GGAGATGCAGGCTCCACTTT
MMP14	Forward: GAGCATTCCAGTGACCCCTC<br>Reverse: ACCCTGACTCACCCCCATAA
MMP2	Forward: GCTTCCAGGGCAATCCTAT<br>Reverse: AACAGTGGACATGGCGGTCT
FSCN2	Forward: AGCCACACAAGTTTCTGCCA<br>Reverse: TGGGGGCCCGACAAAAT
CYCLIN D1	Forward: CGTGGCCTCTAAGATGAAGGA<br>CGGTGTAGATGCACAGCTTCT
IL6	Forward: CAGTTCCTGCAGAAAAAGGCAA<br>Reverse: ATTTGTGGTTGGGTCAGGGG
COX2	Forward: AGAAAACTGCTCAACACCGGA<br>Reverse: GTGCACTGTGTTTGGAGTGG
P21	Forward: GGAGACTCTCAGGGTCGAAAA<br>Reverse: GGCGTTTGGAGTGGTAGAAAT
PAI1	Forward: GCCTCGGTGTTGGCCATGCT<br>Reverse: GGGGGCCATGCCCTTGTCATC
FABP4	Forward: ATGGGGGTGTCCTGGTACAT<br>Reverse: GACGCATTCCACCACCAGTTT
LPL	Forward: CCGCCGACCAAAGAAGAGAT<br>Reverse: TAGCCACGGACTCTGCTACT
PDK4	Forward: TAACACTCTAAGCATAACTAAAGGT<br>Reverse: CACACCATTCCCCATTGTGATT
TGM2	Forward: ATGCGACCCTCCGGGACG<br>Reverse: ATCTGTCACCACATAATTACCT

Table S4. siRNA sequence of PPARγ

**siRNA**	**Sequence (5'-3')**
PPARγ siRNA 1 Sense	GGGCGAUCUUGACAGGAAA(dTdT)
PPARγ siRNA 1 Antisense	UUUCCUGUCAAGAUCGCCC(dTdC)
PPARγ siRNA 2 Sense	GGAAAGACAACAGACAAAU(dTdT)
PPARγ siRNA 2 Antisense	AUUUGUCUGUUGUCUUUCC(dTdG)
PPARγ siRNA 3 Sense	GGAUGCAAGGGUUUCUUCC(dTdT)
PPARγ siRNA 3 Antisense	GGAAGAAACCCUUGCAUCC(dTdT)
